# Genetic predisposition for aggressive behaviour related with dopamine and serotonin pathways – an overview

**DOI:** 10.1080/07853890.2021.1897426

**Published:** 2021-09-28

**Authors:** Cathy Paulino, Alexandra R. Fernandes, Pedro V. Baptista, Cristina Soeiro, Ana Rita Grosso, Alexandre Quintas

**Affiliations:** a Centro de Investigação Interdisciplinar Egas Moniz (CiiEM), Caparica, Portugal; b Laboratório de Ciências Forenses e Psicológicas Egas Moniz (LCFPEM), Caparica, Portugal; c Instituto Universitário Egas Moniz (IUEM), Caparica, Portugal; d Escola Superior de Saúde Egas Moniz (ESSEM), Caparica, Portugal; e UCIBIO, Department of Life Sciences, Faculdade de Ciências e Tecnologia, Universidade NOVA de Lisboa, Caparica, Portugal; f Laboratório de Psicologia Egas Moniz (LabPSI-EM), Caparica, Portugal

## Abstract

**Introduction:**

Aggression is a pervasive condition in human civilisation that sometimes emerge in human actions and consists in physical and verbal actions with the intention of causing harm to others. The term aggression can be divided in two types. Expressive aggression appears by provocation and it is usually driven by anger. Otherwise, instrumental aggression is performed as a premeditated mean to obtain something [1]. Aggressive behaviour is a complex process that involves the interaction between diverse factors, including genetic and environmental factors. The aim of this study is to give an overview of the several studies that have identified genetic alterations that may influence the behaviour of individuals. In terms of aggression, research is focussed on signalling pathways, especially involving dopamine and serotonin [2].

**Materials and methods:**

Searches were made in the PubMed and B-on online databases. Search terms included “Genetic variants” combined with “Aggression” or “Aggressive behaviour”. The search was limited to articles published on or after the 1^st^ of January 2000 and in English-language peer-reviewed journal publications. Papers focussed on genetic variants that did not relate to the dopamine and serotonin pathways were excluded. Further literature sources were identified by following up internal citations and references. After articles analysis, 24 studies were considered in this literature review.

**Results:**

The search for genes involved in impulsivity and aggression traits identified different candidate genes belonging to dopamine and serotonin pathways, of which *MAO-A*, *COMT*, *SLC6A4*, *SLC6A3*, *HTR1A*, *HTR1B*, *HTR2A*, *HTR2C*, *HTR6*, *DRD2*, *DRD4*, *TPH1* and *DBH* genes [3,4]. Results revealed the presence of 18 variants. The majority of these variants were SNPs, except 5 VNTR and 1 InDel.

**Discussion and conclusions:**

Some studies found associations between several genetic variants and aggression. However, in many others such associations were unclear [2,3]. The fusion of genetics and psychology into these studies is essential. Notwithstanding, this literature review showed that all previous studies applied a limited psychological assessment of the subjects, leading to partial characterisation of the problem [4]. As such, it is critical to get a deeper insight into the association between aggressive behaviour and putative genetic factors by redefining the methodological strategies used for the psychological assessment.
